# Paraurethral Leiomyoma: A Rare Entity Requiring Extensive Preoperative Counseling and Prompt Management

**DOI:** 10.7759/cureus.61684

**Published:** 2024-06-04

**Authors:** Deerush Kannan Sakthivel, Meera Ragavan, Sandeep Bafna, Madhav Tiwari, Narasimhan Ragavan

**Affiliations:** 1 Urology, Apollo Hospitals, Chennai, IND; 2 Urogynaecology, Apollo Hospitals, Chennai, IND

**Keywords:** female urethra, urethral disease, obstructive urinary symptoms, urethral leiomyoma, urethral mass

## Abstract

Paraurethral leiomyoma is an exceptionally rare benign smooth muscle tumor adjacent to the female urethra, presenting diagnostic challenges due to nonspecific symptoms like urinary obstruction and dysuria. This case report details the clinical presentation, diagnostic workup, and surgical management of a 45-year-old woman with a paraurethral leiomyoma. Diagnosis involved clinical examination, imaging, and biopsy. The mass was excised via a perineal route without urethral injury, confirmed by histopathology. The patient recovered well, voiding without difficulty postoperatively. This case emphasizes the importance of thorough preoperative counseling, advanced imaging, and multidisciplinary collaboration in managing paraurethral leiomyomas.

## Introduction

Paraurethral leiomyoma is an exceptionally rare benign smooth muscle tumor that arises adjacent to the female urethra. While leiomyomas are commonly found in the uterus, their occurrence in the periurethral region is uncommon and often poses diagnostic challenges due to their rarity and the nonspecific nature of their symptoms [1]. These symptoms can include urinary obstruction, dysuria, and palpable mass, which overlap significantly with more common urological and gynecological conditions [2]. Accurate diagnosis typically requires a combination of clinical examination, imaging, and histopathological analysis [3]. This case report aims to contribute to the limited body of literature on paraurethral leiomyomas by detailing the clinical presentation, diagnostic workup, surgical management, and histopathological findings of a newly diagnosed case, thereby enhancing awareness and guiding future management of similar presentations enhancing awareness and guiding future management of similar presentations.

## Case presentation

A 45-year-old woman without any comorbidities presented to the urology department with obstructive voiding symptoms. She also had a recent history of two episodes of culture-positive urinary tract infections that were managed with antibiotics elsewhere. She did not have any episodes of hematuria or irritative lower urinary tract symptoms. Clinical examination by the urogynecologist revealed a firm mass palpable per vaginal examination, located in the anterior wall of the vagina, with the rest of the examination within normal limits.

**Figure 1:**
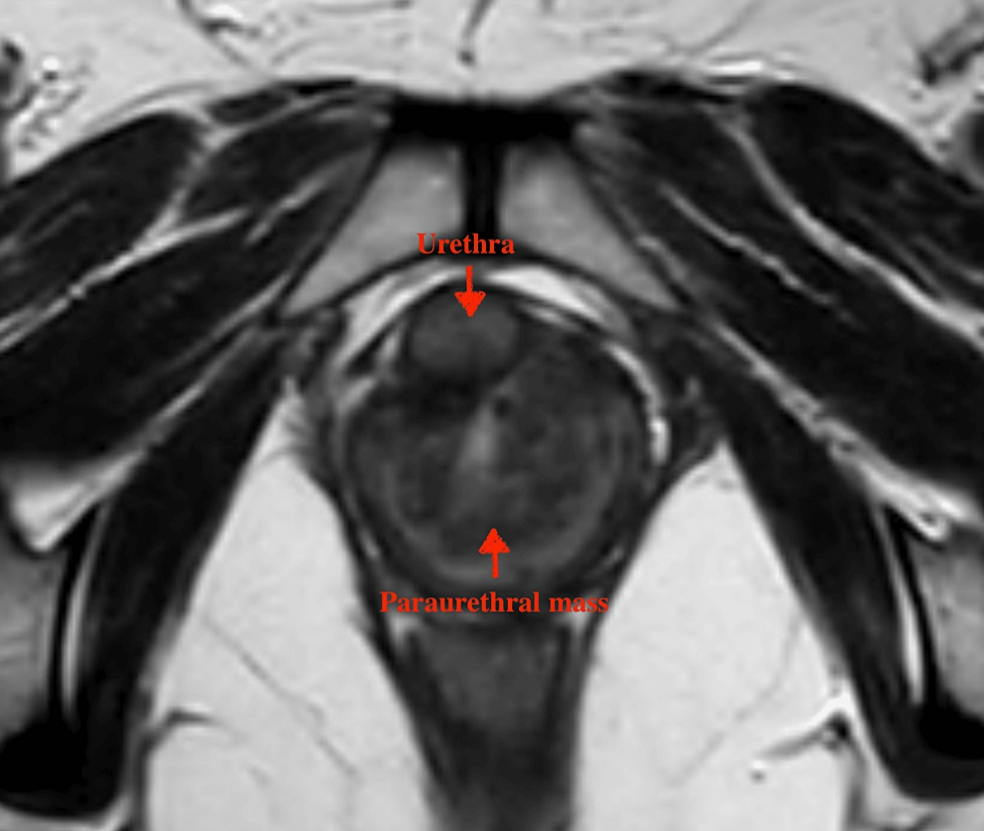
MRI showing the solid paraurethral mass in close relation to the urethra

An ultrasound of the kidneys, ureters, and bladder (KUB) showed a normal-walled bladder with normal upper tracts. All blood parameters were within the normal range, and the urine culture did not reveal any growth. Uroflowmetry revealed an obstructive pattern. In view of the firm mass, a magnetic resonance imaging (MRI) of the pelvis was performed, focusing on the area of interest. The MRI revealed a solid mass, likely of mesenchymal origin (Figure 1).

To proceed with surgery, biopsy evidence was needed, so a transrectal ultrasound-guided biopsy was performed. This confirmed that the mass was closely related to the posterior aspect of the urethra. Six core biopsies were taken, and the biopsy results identified the mass as a leiomyoma.

The patient was counseled about the need for excision and the potential for a minimally invasive abdominal approach if the tumor could not be excised via the perineal route. She was informed about the possibility of urethral injury if the mass invaded close to the urethra and the potential need for a graft in case of intraoperative urethral injury. The possibility of fistula formation and stress incontinence if the mass extended adjacent to the bladder neck was also explained.

**Figure 2:**
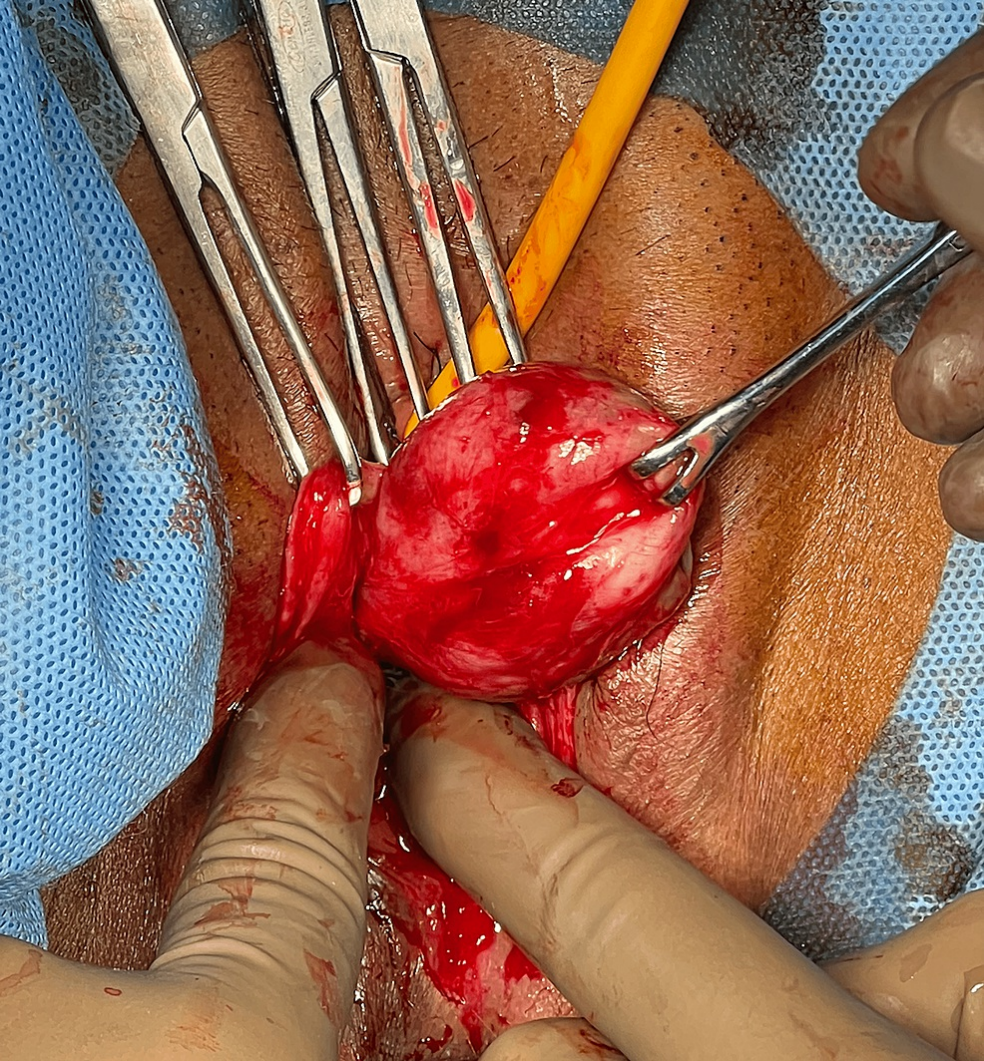
Mass excised with the combination of blunt and sharp dissection

Examination under anesthesia revealed a palpable mass that could protrude upon examination due to the relaxed pelvic muscles. The proximal extent of the mass was well palpable and visible under anesthesia; therefore, the mass was excised via the perineal route (Figure 2). The bladder was catheterized prior to the procedure. A vertical incision was made over the anterior vaginal wall, and the planes were created by blunt and sharp dissection. The mass was excised in toto without any injury to the urethra. Since the mass was dissected with clear planes well away from the urethra, there was no need for a flap cover.

**Figure 3:**
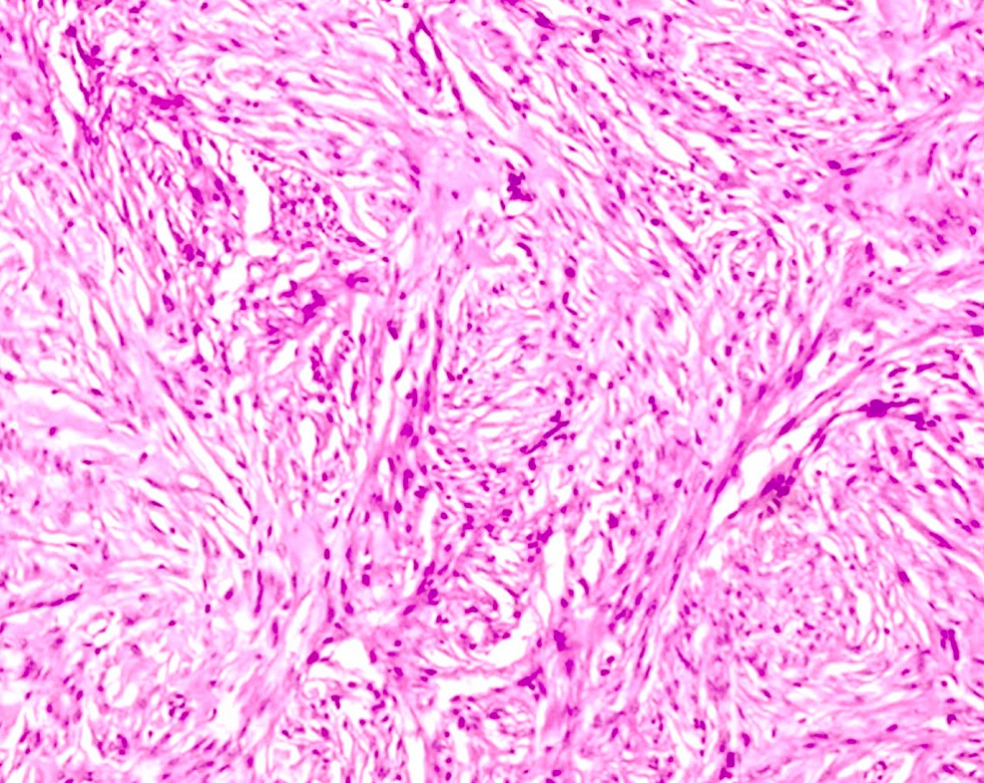
Histopathological image of the final resected specimen showing bundles of smooth muscle cells

The patient was discharged on the first postoperative day with a Foley catheter. The final histopathological diagnosis confirmed paraurethral leiomyoma with all margins clear (Figure 3). The Foley catheter was removed on the fourth postoperative day, and she voided well without difficulty. She is under follow-up every six months and has been doing well without any urinary symptoms or clinical evidence of recurrence.

## Discussion

Paraurethral leiomyomas are exceptionally rare benign tumors arising from smooth muscle tissue near the female urethra. Due to their uncommon occurrence, the literature on the subject is sparse, and each case contributes significantly to the understanding and management of these tumors.

Historically, paraurethral leiomyomas have been reported infrequently, with most information derived from isolated case reports and small case series. Malik and Blake detailed a similar case of a paraurethral leiomyoma, emphasizing the diagnostic difficulties due to overlapping symptoms with more common urological conditions, such as urethral diverticulum and periurethral cysts [2]. They underscored the importance of comprehensive imaging and histopathological confirmation, as clinical examination alone is often insufficient for an accurate diagnosis.

Widia et al. discussed the role of MRI in diagnosing paraurethral leiomyomas, noting its superior ability to delineate soft tissue structures and provide detailed information on the mass's extent and relation to surrounding tissues [3]. This aligns with our case, where MRI was pivotal in identifying the mass's probable mesenchymal origin and guiding the subsequent biopsy and surgical approach.

The surgical management of paraurethral leiomyomas varies depending on the tumor's size, location, and relationship with adjacent structures. Matthew et al. described a case where a leiomyoma was successfully excised via a vaginal approach, similar to the approach used in our case [4]. Their report highlighted the importance of careful dissection and the potential for minimally invasive techniques to achieve complete excision while minimizing complications.

Furthermore, the literature emphasizes the need for preoperative counseling regarding potential complications, such as urethral injury, fistula, and stress incontinence [5]. Our case reinforces this practice, as thorough patient counseling was conducted to prepare the patient for potential outcomes and ensure informed consent.

The literature also indicates that postoperative outcomes are generally favorable when the tumor is completely excised with clear margins. Few cases of recurrence or significant complications have been reported, emphasizing the effectiveness of complete surgical excision [6]. Our patient’s smooth postoperative recovery and lack of complications corroborate these findings.

## Conclusions

Paraurethral leiomyoma is a rare but clinically significant condition that warrants meticulous preoperative counseling and prompt surgical intervention. This case underscores the importance of multidisciplinary collaboration, advanced imaging techniques, and patient education in achieving successful outcomes. Continued vigilance through regular follow-up is essential for monitoring recurrence and ensuring long-term patient well-being.
